# Effects of RF Magnetron Sputtering Power on the Mechanical Behavior of Zr-Cu-Based Metallic Glass Thin Films

**DOI:** 10.3390/nano13192677

**Published:** 2023-09-29

**Authors:** Tra Anh Khoa Nguyen, Nhat Minh Dang, Chi-Hang Lin, Meng-Chieh Lee, Zhao-Ying Wang, Yao-Chuan Tsai, Ming-Tzer Lin

**Affiliations:** 1 Graduate Institute of Precision Engineering, National Chung Hsing University, Taichung 402, Taiwan; khoa.eur@gmail.com (T.A.K.N.);; 2 Aeronautical Systems Research Division, National Chung-Shan Institute of Science and Technology, Taichung 407, Taiwan; 3 ASML Technology Taiwan Ltd., Hsinchu City 300, Taiwan; 4 Department of Bio-Industrial Mechatronics Engineering, National Chung Hsing University, Taichung City 402, Taiwan; yctsaii@dragon.nchu.edu.tw; 5 Industrial and Smart Technology Program, Academy of Circular Economy, National Chung Hsing University, Nantou 540, Taiwan

**Keywords:** metallic glass thin film, Zr-based metallic glass, amorphous structure, creep, residual stress

## Abstract

Zirconium-based metallic glass films are promising materials for nanoelectronic and biomedical applications, but their mechanical behavior under different conditions is not well understood. This study investigates the effects of radio frequency (RF) power and test temperature on the nanostructure, morphology, and creep behavior of Zr_55_Cu_30_Al_10_Ni_5_ metallic glass films prepared by RF magnetron sputtering. The films were characterized by X-ray diffraction and microscopy, and their mechanical properties were measured by a bulge test system. The results show that the films were amorphous and exhibited a transition from noncolumnar to columnar morphology as the RF power increased from 75 W to 125 W. The columnar morphology reduced the creep resistance, Young’s modulus, residual stress, and hardness of the films. The creep behavior of the films was also influenced by the test temperature, with higher temperature leading to higher creep strain and lower creep stress. The findings of this study provide insights into the optimization of the sputtering parameters and the design of zirconium-based metallic glass films for various applications.

## 1. Introduction

Thin films are increasingly being used across a range of different applications. The development of thin films as a replacement for bulk materials has made them an indispensable choice for Micro-Electromechanical Systems (MEMSs). As a kind of solid metallic material, Bulk Metallic Glasses (BMGs) have obtained a distinct properties list based on high fracture toughness, high elastic strain limit, superior mechanical strength, and lower Young’s modulus [1]. Since the advancement of science and technology and the vigorous development of MEMSs, the number of their applications has been increasing significantly [2,3,4,5]. Because of the excellent mechanical properties of metallic glass, there are many studies on the application of metallic glass in MEMSs [6,7,8,9,10,11].

Compared with the crystalline structure, metallic glass is a type of solid metallic material that possesses a unique set of properties, such as high corrosion resistance, high strength, and excellent elastic strain limit, that makes it highly desirable for various applications [12]. In recent years, researchers have focused on developing different types of Bulk Metallic Glasses (BMGs), including Zr-based, Pd-based, Fe-based, and Au-based alloys [13,14,15,16,17,18,19,20,21]. Among these, Zr-based alloys have gained significant attention due to their exceptional properties, such as high hardness, high wear and corrosion resistance [22,23,24], antimicrobial activity, biocompatibility [23,24,25,26], good antibacterial activity [27,28,29], excellent micro-molding ability, stability at a glass transition temperature (Tg), broad subcooled liquid region of about 100 K [13,30,31], and high catalytic behavior [32]. These properties make Zr-based BMGs suitable for use as parts in Micro-Electromechanical Systems (MEMSs). One of the key issues that affect the reliability and durability of MEMS parts is material creep. Creep behavior refers to the time-dependent deformation of a material under a constant load or stress. In 2005, Tuck et al. [31] studied the creep behavior of polysilicon, one of the most widely used materials in the MEMS field. Since then, researchers have continued to investigate the creep behavior of different materials used in MEMSs to better understand the impact it has on the life and durability of these parts. Barbato et al. [33,34] examined the creep effects in a gold RF MEMS switch and utilized temperature as a means to estimate the device’s longevity. In addition, Hsu [35,36] employed a highly accurate capacitance-sensing setup to characterize nickel RF-MEMS devices and found that creep deformation predominantly governs the device’s behavior under a specific bi-state bias condition. In light of the unique properties of Zr-based BMGs, the development of Zr-based alloy thin films has received significant attention in recent years. These properties make Zr-based BMGs suitable for MEMS applications. Therefore, studying the creep behavior of Zr-based BMGs is of great importance to ensure the reliability and durability of MEMS parts made of these materials.

Metallic Glass Thin Film (MGTF) manufacturing methods include evaporation, electroplating, sputtering, etc. [37]. In this study, magnetron sputtering, one of the main ways to produce MGTF, was employed. Since bulk metallic glass is still affected by factors related to size, cost, and brittleness, the relative advantages of metallic glass films that make them suitable for companies to actively invest in the research and commercialization of MGTF-related applications include (1) having a thick material size, leading to the inside and outside of the material having different cooling rates [38], which could easily result in an uneven amorphous structure; this significant enhancement in characteristic specimen sizes has been primarily achieved through the utilization of multi-component composition design. Accordingly, many metallic glass (e.g., Zr-, Cu-, Ti-based, etc.) systems with sizes in excess of the value have been reported [39]; (2) the ability of the composition ratio of alloying elements or process parameters to be changed; and (3) the ability of the mechanical properties (hardness, wear resistance, etc.) of the metallic glass to be adjusted through analysis by High-power impulse magnetron sputtering (HiPIMS) and DC magnetron sputtering at the same average power. Utilizing a single Zr_60_Cu_25_Al_10_Ni_5_ target [40], Zhang et al. [41] investigated the impact of adding Fe on the mechanical properties of metallic glass and found that the alteration in the distribution and amount of free volume within the metallic glass sample was the primary factor responsible for the observed improvement in its mechanical performance. Inoue et al. [42] conducted a detailed analysis on the influence of strain rate on the deformation behavior of Zr-Cu-Al-Ag metallic glasses. The field of biomedicine is highly particular about the mechanical properties of the materials used for medical appliances; since MGTF has excellent mechanical properties, it actively invests in experiments and researches its applications in related fields. The Zr_55_Cu_30_Al_10_Ni_5_ alloy represents a prominent metallic glass alloy, renowned for its multifaceted and commendable physical, chemical, and mechanical attributes. In contrast to the Zr-Cu alloy systems, the incorporation of a minor proportion of aluminum (Al) within the Zr-Cu-Al metallic glasses (MGs) manifests a rapid and substantial augmentation in the proliferation of Floppy Inclusions (FIs) and the magnitude of their spatial interconnectedness. Notably, the incorporation of aluminum (Al) into this alloy matrix plays a pivotal role in augmenting the Glass-Forming Ability (GFA) of the material and bolstering its thermal resilience [43,44,45,46]. This enhancement in glass-forming ability is rooted in the unique atomic interactions and structural rearrangements facilitated by the presence of aluminum (Al) atoms within the alloy composition. The existing body of literature primarily focuses on alloys characterized by a singular Al content of 10%. These studies [47,48,49] collectively emphasize the pronounced influence of this specific Al composition in mitigating kinetic processes, thereby enhancing the glass-forming ability of the alloys. Furthermore, it is evident from these investigations that the inclusion of aluminum leads to augmented stability within the atomic structure of the alloy. Furthermore, the inclusion of nickel (Ni) in the Zr_55_Cu_30_Al_10_Ni_5_ alloy serves as an additional catalyst for elevating its glass-forming potential and reinforcing its thermal stability. The nickel contribution to this alloy configuration is predicated upon its capacity to induce structural modifications that further fortify the alloy’s capacity to assume an amorphous, glassy structure [50]. In a study by Zhu et al. [51], the addition of Ni to the ZrCuAl alloy resulted in a notable decrease in both hardness and Young’s modulus, making ZrCuAlNi 13% and 19% lower, respectively, compared to ZrCuAl. Moreover, Ni addition increased the material’s stored energy and the presence of loosely packed regions in its microstructure. Consequently, the combined influence of Al and Ni emerges as a critical factor in the endeavor to enhance the glass-forming capability and thermal endurance of the Zr_55_Cu_30_Al_10_Ni_5_ metallic glass alloy [52]. These alloying elements collaborate synergistically to create a robust alloy system with a wide array of applications in diverse industrial and technological domains.

The interest in bulge testing rose when the development of the semiconductor industry made micromachining methods widely available. To characterize thin films, the bulge testing method is primarily used, and the mechanical properties of thin films are determined via mathematical and analytical approaches. These methods have been well adopted for rectangular thin films to calculate the stresses and strains at the central point without the dependency on their properties. There is a dependence of the equilibrium conditions on the curvatures generated at the peak of deflection since the curvatures are generated at this point [53]. There are a number of shapes that can be used here to represent the kinematics of the bulged surface, including a spherical cap and cylindrical shapes. Vlassak and Pratt [54] were among the first to investigate rectangular membranes, and this improvement in their theory allowed a wider variety of applications.

For multilayered thin films, creep behavior at constant load levels can serve as the basis for the design of such thin-film materials during their functional applications. Besides affecting their electrical conductivity, time-dependent creep may also affect their optical properties, like light reflection or transmission, and their electrical conductivity. Therefore, the creep behavior of alloy thin films would have a significant effect on their performance.

As far as we know, only a few studies have focused on investigating RF magnetron-sputtered Zr-based metallic glass alloys and mostly examined only a few material properties in a specific range of the elemental composition [55,56]. It is a widely used method for the synthesis of metallic thin films. In recent years, there has been a growing interest in the use of plasma synthesis for the production of nanomaterials [57,58,59]. This technique involves the use of plasma to create highly reactive species that can be used to synthesize nanomaterials with unique properties. The plasma particles, including ions, electrons, excited and neutral particles, and radicals, are at different temperatures, which makes it possible to synthesize a wide range of nanomaterials [60,61]. In this study, we selected a Zr_55_Cu_30_Al_10_Ni_5_ alloy as the experimental material based on its exceptional GFA [62]. Notably, this alloy composition lacks toxic or noble elements. The research involved the continuous fabrication of Zr_55_Cu_30_Al_10_Ni_5_ thin-film alloys, utilizing varying process parameters under three different radio frequency (RF) source power conditions. XRD, Scanning Electron Microscopy (SEM), and Energy Dispersive X-ray Spectroscopy (EDS) investigated the nanostructure and surface morphology of Zr-based MGTF. The mechanical properties of samples were carried out, such as Young’s modulus, residual stress and hardness by bulge test, and nanoindentation. Furthermore, the bugle test will be provided with additional heating in order to facilitate an analysis of the creep behavior of the MGTFs.

## 2. Materials and Methods

### 2.1. Preparation of the Specimens

**Figure 1:**
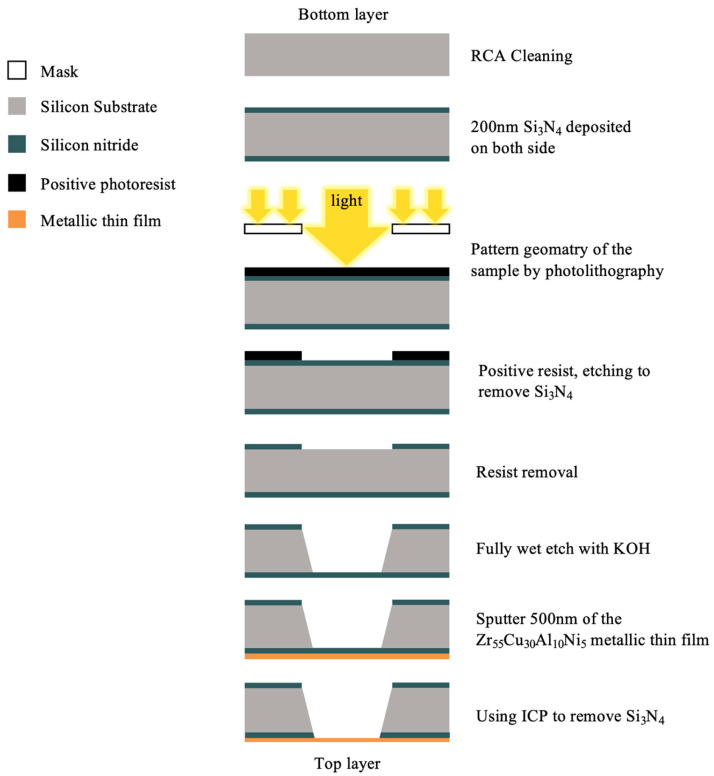
The sequence of the fabrication process.

**Figure 2:**
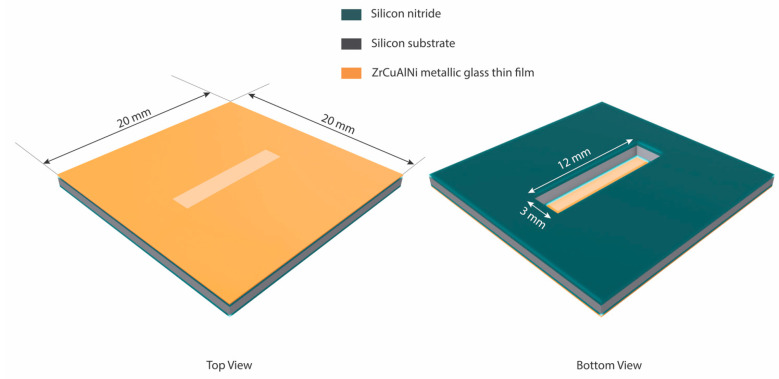
Size and dimensions of the bulge sample.

The process of sample fabrication is shown in Figure 1. The <100> silicon wafers used were 100 mm in diameter, polished on both sides, and had a thickness of 200 μm. First, the silicon wafer was cleaned with Radio Corporation of America (RCA) cleaning, a process used to remove organic residue from silicon wafers. Second, 200 nm Si_3_N_4_ thin films was deposited using a thermal Low-Pressure Chemical Vapor Deposition (LPCVD) on both sides of the silicon substrate. A thin layer of photoresist was deposited on wafers by the photolithography technology. Third, the photoresist was removed, and the cover layer was etched by an Inductively Coupled Plasma (ICP) in order to remove the SiNx layer of the pattern. An ICP Etching System was utilized to apply a silicon nitride film on the unprotected areas of the wafer, where a positive photoresist was absent. This approach offers the advantage of achieving non-isotropic etching, resulting in higher resolution of the etched films. Additionally, reactive ion etching, a type of dry etching, was employed. The structure of the system consists of parallel electrode plates, with the upper electrode functioning as a gas distribution plate. The etching gas is evenly distributed into the cavity from the top. The RF field generated by the equipment ionizes the gas molecules, transforming them into a plasma state. The plasma is then influenced by the bias voltage field, causing it to be directed towards the surface of the test piece. Consequently, the plasma reacts with the film surface, leading to its removal. In this specific experiment, CF_4_ gas was selected as the etching gas. To create a floating membrane from the SiN_x_ layer, we used wet etching as the next step in the process, leaving a full free-standing silicon layer in the rectangular membrane openings. Thereafter, the base pressure of the system chamber was set at 5 × 10^−6^ Pa, and the working pressure was set at 1 Pa in order to achieve the desired result. Then, 500 nm of zirconium-based MGTFs was deposited onto the specimen by using an RF magnetron sputtering system (Magnetron Sputtering Systems, AdNaNoTek, SPUTTER-3 (SPEED), New Taipei City, Taiwan) with a 3-inch (76.2 φ) sputtering target alloy of composition Zr-Cu-Al-Ni (55:30:10: 5 atm%). These kinds of specimens were made by adjusting the power supply wattage during the coating process. Finally, the etching was performed using ICP to remove the window part of the silicon nitride on the membrane; in practice, three kinds of specimens were produced, and Table 1 illustrates a list of the compositions. The membrane geometry of the bulge test samples was 3 × 12 mm^2^ rectangular (shown in Figure 2).

### 2.2. Methodology

The plane–strain bulge test at room temperature is an effective method for measuring the mechanical behavior of thin films, such as Young’s modulus, and can be utilized to calculate the residual stresses $\sigma_{0}$ [63]. It has been reported in the literature [64] that the Zr_55_Cu_30_Al_10_Ni_5_ metallic glass exhibits a Poisson’s ratio (represented by the symbol v) equal to 0.369. To determine the residual stress in the film, the unloading portion of the stress–strain curve was analyzed with a strain value of zero. According to Vlassak et al. [54], the lateral stress, $\sigma_{bulge},$ and strain, $\varepsilon_{bulge}$, of a membrane under applied pressure are proportional to its deflection, H_T_. The pressure versus bulge height and the stress versus strain of the specimens are the stress of the film from the pressure-displacement data recorded during the experiment, which was calculated using Equation (1):

(1) σbulge=2HT23D22E1−ν2+σ0

Meanwhile, the true principal strains were calculated using Equation (2):

(2) εbulge=2HT23D22

where:

*H_T_* is the bulge height of the specimen at time *T*.

*D* is the width in the center of the membrane.

*E* is Young’s modulus.

In this study, the equivalent creep strain was calculated using the model that is often used to describe creep deformation, which, based on the power law form of the equivalent creep strain rate also known as the Bailey–Norton law [65], is a modification of Norton’s law [66] that takes into account the effect of temperature on the creep behavior of materials, and is widely used to explain time-dependent deformation at elevated temperatures under constant stress [67].

When using the Bailey–Norton creep law to obtain equivalent creep strain, a Prony series was used to fit the linear viscoelastic behavior of the tested material [21]. The Prony series equation takes the form:

(3) εT=ε0+∑i=1Nεi1−e−TτiG

where:

$\varepsilon_{i}$ and $\tau_{i}^{G}$ are the Prony series coefficients and the corresponding retardation times.

$\varepsilon_{0}$ is the instantaneous elastic strain.

In this case, since the material is assumed to exhibit low temperature dependence, the retardation time $\tau_{i}^{G}$ was assumed to be constant for all models. The coefficients $\varepsilon_{i}$ are determined by fitting the Prony series equation to the equivalent creep strain data obtained from the Norton creep law model. It is worth noting that while the Norton creep law model assumes that creep behavior is temperature dependent, it can still be used to obtain creep data for materials that exhibit low temperature dependence. In these cases, the Prony series can be used to fit equivalent creep strain data to describe the linear creep behavior of the material [66,67,68,69].

In addition, thin-film hardness and Young’s modulus were determined using a Nanomechanical Test System (BRUKER, Hysitron TI 980 Nanoindenter, Billerica, MA, USA). The properties were determined using the Oliver and Pharr method [65]. This method is essential in determining the contact area in nanoindentation with depth-sensing instrumentation. It enables the computation of the hardness using Equation (4):

(4) $$H=\frac{P_{max}}{A}$$

One can accurately factor in the impact of indenters on load-displacement behavior by defining Young’s modulus using Equation (5). The commonly used equations in nanoindentation for the calculation of modulus are as follows:

(5) $$\frac{1}{E_{r}}=\frac{\left(1-v^{2}\right)}{E}+\frac{\left(1-v_{i}^{2}\right)}{E_{i}}$$

where:

The values of *E* and *ν* for the thin film and the indenter are $E_{i}$ and *ν_i_*, respectively.

## 3. Experimental Details

**Figure 3:**
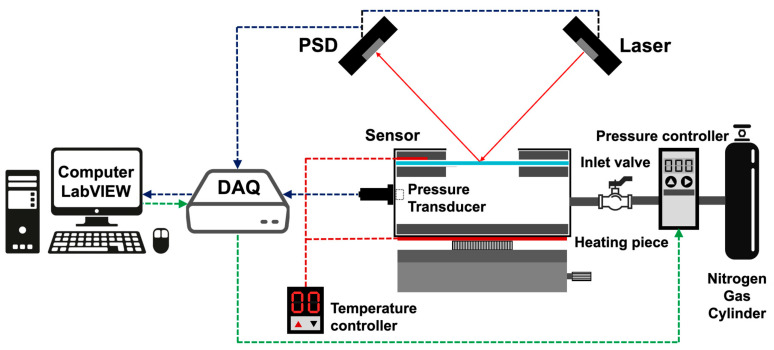
Schematic diagram of the bulge system.

**Figure 4:**
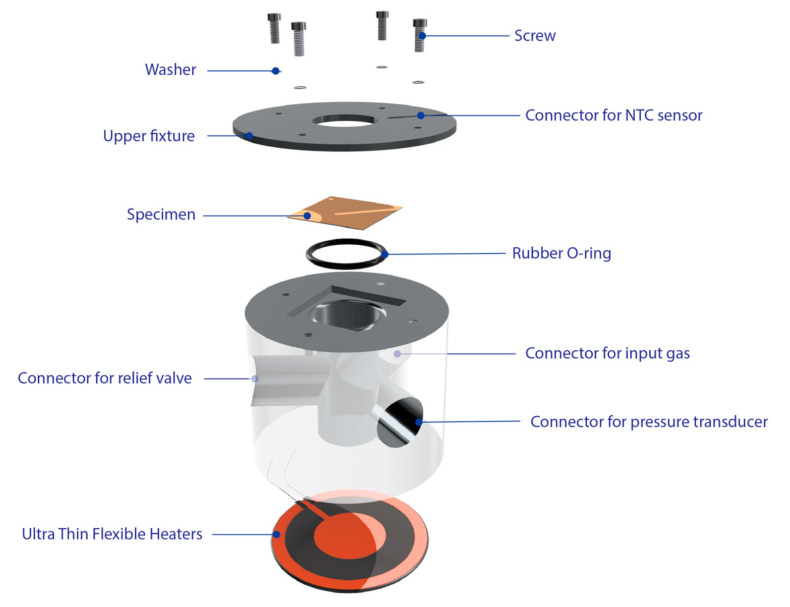
Illustration depicting the chamber setup.

In order to investigate the mechanical properties of thin films, bulge testing was conducted to examine their mechanical properties. The sample is mounted on a pressure chamber and is pressurized with nitrogen gas controlling the stress. The experiment optimized the hardware of the bulge system used in a previous study [70,71] with the addition of a heating system. The experimental setup of the bulge system is shown in Figure 3. This system comprised the following: Data Acquisition (DAQ, National Instruments, USB-6341-BNC, Austin, TX, USA) as the signal source, a pressure controller (Digital electro-pneumatic regulator, CKD, EVD-1500-008AN, Komaki, Aichi, Japan), a custom vacuum chamber, a slide rail table, and a heating system. Data are collected by the DAQ during signal acquisition, then received by the pressure transducer (JETEC, JPT-131S, Taichung, Taiwan). To perform the experiment, the position sensor (Quanzhou Optoelectronics, Position Sensing Detector, PSD, Quanzhou, China) was used as the measurement device. This has an excellent sensitivity and a high acquisition rate with displacement changes, which is suitable for the measurement of small deformations. In addition, it has a single-point laser light used to irradiate the surface of the film, which is then reflected in the area above the PSD. Meanwhile, the thin film was deformed by the applied pressure changing the height and deforming the membrane of the sample. The PSD returns the voltage signal and uses the LabView graphics control software to write a program to convert the pressure and signal to the stress, and its corresponding strain values were calculated. The pressure is controlled by changing the voltage signal with the output control end. The heating system is controlled by digital temperature and time control devices (KLC, DTC-A1045B-150, Taichung, Taiwan) to adjust the temperature of the Ultra-Thin Flexible Heaters (KLC, TSA(C)050d006hR28, Taichung, Taiwan) at the bottom of the cavity, and the actual temperature is measured by an NTC sensor in contact with the sample. A cavity is designed to accommodate the gas pressure to pressurize the sample, and a pressure sensor interface, air inlet, and air outlet are designed on the side of the cavity to ensure the pressurization process. To ensure no leakage of air pressure, the O-ring groove is designed on the carrier to completely seal the test specimen and the cavity. A detailed description is given in Figure 4.

The experimental procedure includes the experimental setup, sample preparation, and thin-film analysis. The experiment uses a flexible ultra-thin electric heater as the heating source; the reference temperature can reach 210 °C, the test specimen is heated using a digital temperature controller, and the NTC sensor is used to measure the surface temperature of the sample. Due to the thicker cavity of the stage, the temperature rise of the heating plate attached to the bottom layer is relatively slow. In order to increase the power of the heating pad and enhance the heating effect, an autotransformer can be used to increase the voltage value of the heating plate.

**Figure 5:**
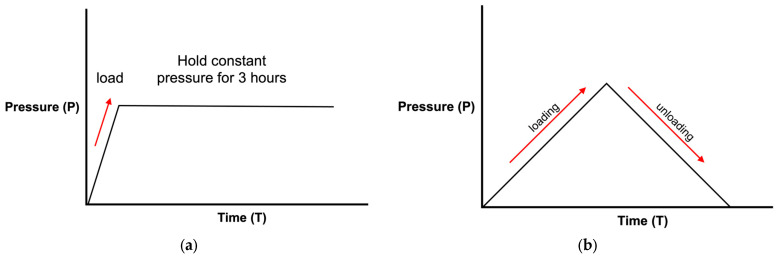
Schematic diagrams of the loading/unloading test (**a**) and creep test (**b**) as performed in the bulge test.

To observe the mechanical properties of the thin film, including the Young’s modulus, residual stresses, and hardness and crystal structure of the ZrCuAlNi thin films, samples were made in three sets with different process parameters by changing the RF power at 75 W, 100 W, and 125 W. The position sensor was used as the measurement device. The experiment measures the bulge height of the thin film versus the pressure by adjusting the inlet pressure using a computer-controlled pressure gauge via a LabView-based program. Additionally, the thin film was deformed by the applied pressure, which changed the height of the thin film and resulted in the deformation of the membrane of the sample. According to this bulge test setup, the stress and deformation of the film were observed by the pressure and bulge height using Equations (1) and (2), as shown in Figure 5a.

For the creep test, the samples were heated to different temperatures (80 °C, 100 °C, and 120 °C), and the strain of the metallic glass film was measured after the thermal equilibrium was reached. The temperature sensor was installed on top of the flat part of the sample, which detected the actual temperature of the surface of the thin film. The electric heating sheet was utilized to heat the bottom of the cavity through conduction to the upper sample to maintain the required temperature and reach an equilibrium state. The intake pressure was maintained and remained constant with the digital proportional control valve. In this study, the holding times for the creep measurements were maintained for three hours, as shown in Figure 5b; the strain value was recorded throughout the experiments.

In addition, the ZrCuAlNi thin-film hardness and Young’s modulus were determined using a Nanomechanical Test System (BRUKER, Hysitron TI 980 Nanoindenter, Billerica, MA, USA). By pressing down on the diamond probe, the surface of the sample is deformed. The probe penetration depth of the displacement sensor can be matched with the load–depth curve obtained from the measurement process using the Oliver and Pharr method [21]. The surface morphology and cross-sectional structure changes of the films were evaluated by field emission SEM (JEOL JSM-7800F, Akishima, Tokyo, Japan). The SEM resolution can reach as high as 0.7 nm (15 kV) and 0.7 nm (1 kV). To prepare the samples for cross-sectional SEM, a series of rigorous steps were undertaken. Initially, the thin films were carefully cut perpendicular to the surface, ensuring that the resulting cross-sectional area was free from any significant deformations or structural irregularities. This was accomplished using precision cutting instruments, which were operated by experienced technicians to avoid any damage to the films during the cutting process. After obtaining the cross-sectional samples, a thin layer of conductive material was deposited on the surface to enhance their conductivity and facilitate SEM imaging. The conductive layer was applied using a specialized sputtering system, which was configured to ensure that the coating was uniform and free from any defects or impurities. This step was essential to minimize the effect of charging during SEM imaging, which can significantly reduce the quality and accuracy of the obtained images. Overall, great care was taken in the preparation of the cross-sectional samples to ensure that they were free from any artifacts or distortions that could impact the analysis of the mechanical and crystal structure of the ZrCuAlNi thin films. These efforts enabled us to obtain high-quality SEM images that revealed the fine details of the film structure and enabled us to perform accurate measurements.

In addition, after the film was deposited, the X-ray EDS installed on the SEM was utilized to analyze the nanostructure element measurement ratio of the ZrCuAlNi thin metallic glass film sample. Grazing Incidence X-ray Diffraction (GIXRD, BRUKER D8 Discover, Billerica, MA, USA) of the Department of Materials Science and Engineering, National Chung Hsing University, was used to determine the crystal structure. The X-rays emitted use a slight incidence angle, and the substrate’s penetration is limited, so it is suitable for studying the crystalline phase of thin films. Using copper as the target material, the characteristic wavelength is 1.5406 A, the working voltage is 40 kV, the working current is 40 mA, the diffraction speed is 2°/min, and the diffraction angle (2θ) is analyzed at 20~80°. After analysis by XRD analysis, the diffraction intensity is compared with JCPDS (Joint Committee of Powder Diffraction Standard card), with a standard pattern to assist manual comparison, and X’Pert High Score Plus software is used for comparison to judge the quality of material in all directions accurately.

## 4. Results

### 4.1. EDS Surface Composition Analysis

**Figure 6:**
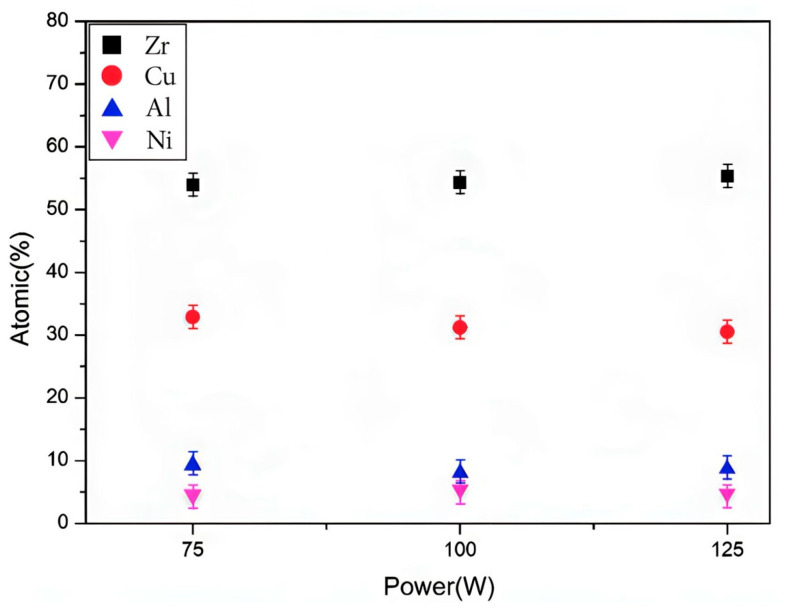
Element ratios of ZrCuAlNi thin metallic glass films plated with different power.

In this study, the composition of the thin-film sample was analyzed using EDS to verify its consistency with the target Zr_55_Cu_30_Al_10_Ni_5_ composition, as shown in Table 2. The EDS analysis revealed the presence of Zr, Cu, Al, Ni, and other elements in the sample. The characteristic peaks of the identified elements were clearly visible in the sample, indicating that the sample’s composition was consistent with the target. The EDS analysis results showed that the element ratio values obtained from the EDS spectra of specimens in Figure 6 were not significantly different from the target composition, confirming the consistency of the sample with the target composition Zr_55_Cu_30_Al_10_Ni_5_.

Regarding the strong line between 1.7 and 1.8 keV in the EDS patterns (Figure 6), it is identified as the Si_3_N_4_ substrate. It is possible that the Si_3_N_4_ substrate contributes to the analysis, which could lead to an overestimation of the Si content in the sample. Therefore, the analysis of the EDS spectra should take into account the contribution of the substrate to the results. Therefore, the EDS analysis performed in this study provided valuable insights into the composition of the thin-film sample, verifying its consistency with the target composition. The analysis also highlighted the importance of considering the substrate’s contribution to the EDS spectra to obtain an accurate composition analysis.

### 4.2. XRD Analysis

**Figure 7:**
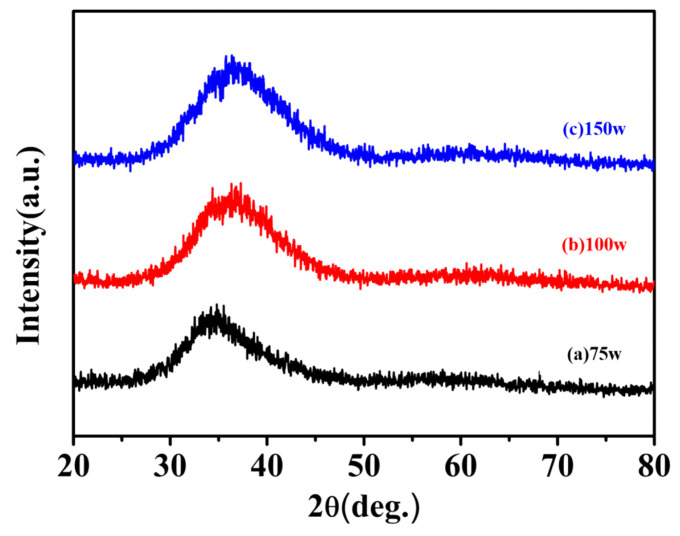
The XRD patterns show the diffuse hump for the amorphous ZrCuNiAl films with sputtering power at 75 W, 100 W, and 125 W.

**Figure 8:**
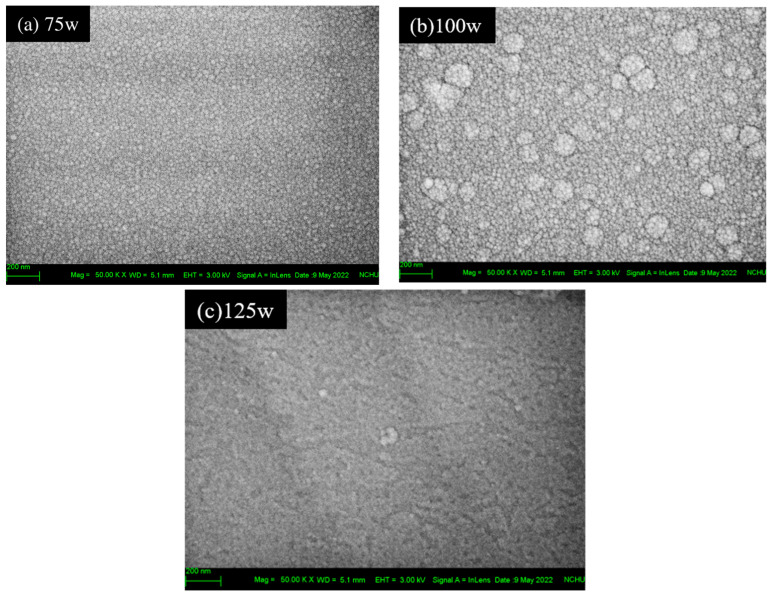
Surface morphology of the samples with different coating powers: (**a**) 75 W, (**b**) 100 W, and (**c**) 125 W.

The XRD analyzer was utilized to obtain the crystal phase analysis diagram and to judge the amorphous form of the Zr-based metallic glass by the generation of diffraction peaks. As shown in Figure 7, the irradiation range was between 20° to 80° under different sputtering power wattages. XRD measurements were conducted, and the findings are presented in Figure 8. No discernible diffraction peaks from crystalline phases were observed in the XRD patterns. Instead, the broad centers of gravity were found to be situated at approximately 2θ ≈ 30° to 45°, a typical amorphous structure with a steamed broad peak that disappeared with the increase in the angle, which means that it contributed to the excellent glass-forming ability [72,73]. There was no characteristic diffraction peak that appeared with the increase in the coating power; the absence of sharp peaks suggests that there is no long-range order in ZrCuAlNi films, indicating the glassy state of the ZrCuAlNi films, so it can be deduced that it is an amorphous structure, which agrees with the literature [74]. It can be seen from the EDS composition analysis that the difference in the element ratio of the film was not large, and it was not caused by the different element ratios. Therefore, it can be judged that the cause of this deviation was the increase in the sputtering power, which increased the kinetic energy of the atom in the internal stress, resulting in the offset phenomenon.

### 4.3. FE-SEM Surface Morphology and Nanostructure Observation

Figure 8 illustrates the changes in the surface morphology of thin films during the coating process under different wattages, and the thickness of the films was measured using field emission scanning electron microscopy (SEM). Specifically, Figure 8a–c represent films coated under power levels of 75 W, 100 W, and 125 W, respectively. The SEM images reveal that the film surface coated at 75 W had small particles (20–24 nm), and the atomic arrangement was disordered. As the coating power increased to 100 W, the particles started to come closer to each other and gradually merged into a group with a size up to 120 nm. Finally, at 125 W, an amorphous ZrCuAlNi thin film with an apparently randomly oriented surface morphology was observed. These results suggest that an increase in power leads to an increase in the atomic kinetic energy, which results in the formation of packed irregular amorphous structures. It is widely believed that surface morphology and uniform film quality have a positive impact on corrosion resistance [75] and good plasticity [11,76]. Therefore, these results suggest that tuning the coating power during the film deposition process can be a practical way to achieve the desired surface morphology and film quality, which, in turn, can lead to improved properties of the material.

**Figure 9:**
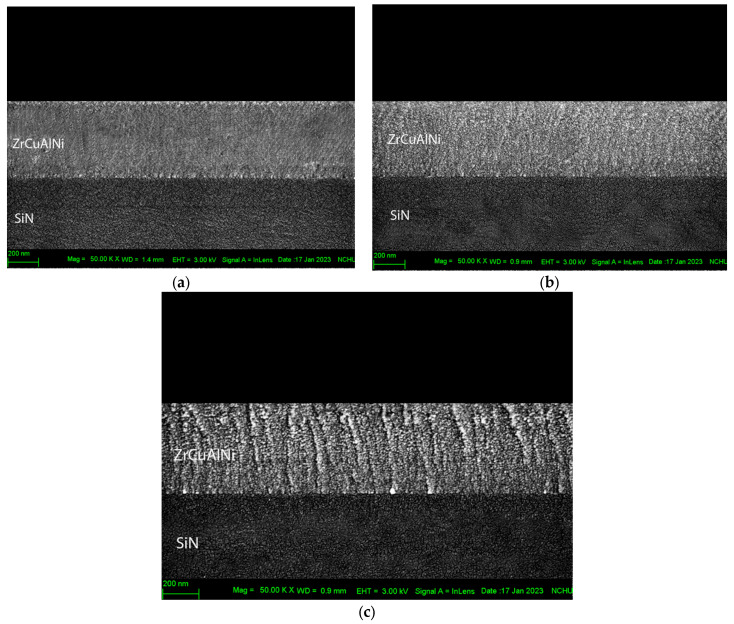
Cross-sectional structure diagram of different coating powers: (**a**) 75 W, (**b**) 100 W, and (**c**) 125 W.

The surface morphology of the ZrCuAlNi layers has been investigated by SEM cross-sections (see Figure 9), and the results suggest that it is strongly affected by the RF power during the sputtering process. It is well known that sputter-deposited film exhibits characteristic columnar growth [77], and in the SEM cross-section images, the changing morphology of the film with RF power can be clearly observed. In the SEM cross-section image of the amorphous ZrCuAlNi thin-film metallic glass, a columnar nanostructured morphology is observed at 125 W. Interestingly, at 75 W and 100 W, the formation of non-column grains and the presence of small equiaxed grains (<5 nm) are evident. As the RF power increases, reaching 125 W, larger columnar structures (20–40 nm) are formed. It has been found that an increase in RF power leads to an elevated flux of particles with higher kinetic energy reaching the surface per unit time. These atoms possess greater mobility on the substrate, resulting in a larger column size, which restricts diffusion and leads to the growth of more islands simultaneously, yielding columns [78].

Despite the potential for a slight increase in atomic mobility at high RF power levels, it is insufficient to induce crystallization in this thin-film coating system. Therefore, it can be concluded that the morphology of the ZrCuAlNi layers is strongly influenced by the RF power used during the sputtering process, and that increasing the power leads to the formation of an irregular amorphous structure of the film. This effect is due to the higher kinetic energy of the atoms that reach the surface, which results in increased mobility and column growth. However, it is important to note that the increase in atomic mobility at high RF power levels is not enough to cause crystallization in the thin-film coating system.

### 4.4. Thin-Film Mechanical Properties

**Figure 10:**
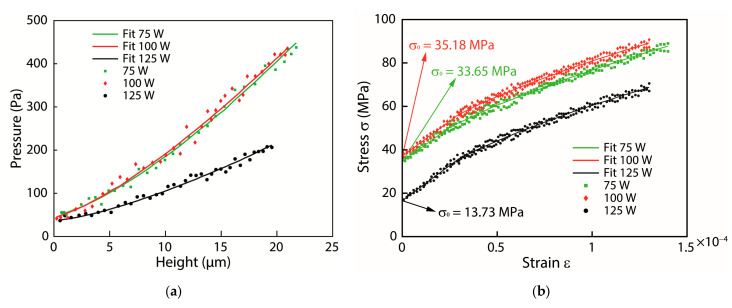
(**a**) Pressure-height curves and (**b**) stress-strain curves of samples diagram.

During the bulge test, the stress and deformation of the film were examined by measuring the pressure and bulge height. The results were studied for three different samples with different RF powers, and the graphical representation of the observations was obtained and illustrated as the pressure–height curves in Figure 10a. The stress–strain curves were also calculated from the pressure–height data, as shown in Figure 10b. In addition, a polynomial curve fitting was applied to these data to aid in the interpretation of the results. The results are useful in determining the mechanical properties of the film and can aid in the optimization of the deposition process to enhance film performance.

Table 3 provides a summary of the mechanical properties of the tested films. The Young’s coefficient of the zirconium-based metallic glass Zr_53_Cu_30_Ni_9_Al_8_Si_0_._5_ was measured to be around 80 GPa, which is consistent with the value reported by Lee [79]. The Young’s modulus obtained from the nanoindentation test was found to be in close agreement with the data obtained from the bulge system. It was observed that as the sputtering power increased from 75 W to 125 W, the Young’s modulus and hardness of the films decreased from 84.25 GPa to 78.65 GPa and from 6.29 GPa to 5.62 GPa, respectively. Similar results have been reported in other studies [80]. These findings suggest that the Young’s modulus and hardness of the zirconium-based metallic glass decreased with increasing sputtering power. The residual stresses of samples 1 (75 W) and 2 (100 W) are similar, but reduced drastically with increasing sputtering power to 125 W. This trend in the influence of sputtering power on residual stress in films is consistent with the findings reported by Wang [81]. To ensure accuracy, the measurement error for samples averaged measurements for each case, as shown in Table 3. We used five homogeneous samples for each power case, and the composition of the samples was consistent throughout. We acknowledge that the heterogeneity of the samples can affect the measurement error, and we took steps to minimize this effect by selecting homogeneous samples and carefully measuring the elemental ratios. Therefore, measurements for each sample ensure that the results are reliable and statistically significant.

### 4.5. Creep Responses

In the creep test, the effect of changes in temperature and time on the strain of the metallic glass film were observed. The temperatures on the three sets of samples were set at 80 °C, 100 °C, and 120 °C, respectively, and the voltage was kept constant through a digital control proportional valve, so that the gas was maintained in the elastic range of the material and the flow rate was fixed to the cavity. The test time lasted for 3 h.

**Figure 11:**
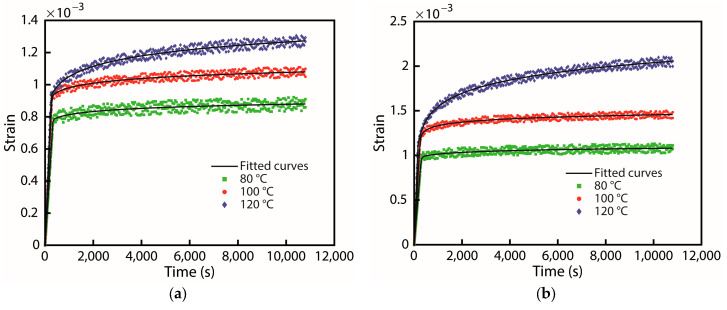

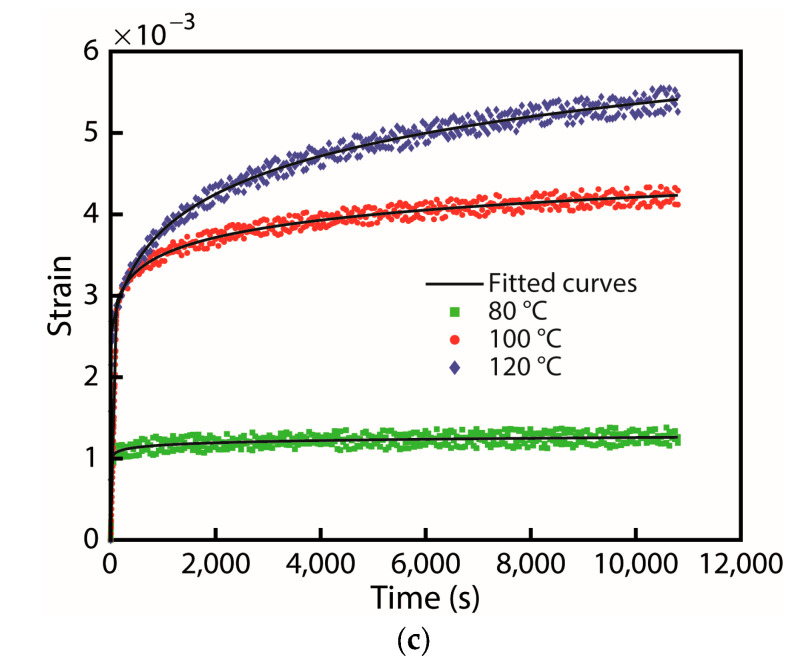
Creep test diagram of different specimen types at (**a**) 75 W, (**b**) 100 W, and (**c**) 125 W.

At a constant testing temperature and applied stress, creep is the time-dependent strain response of constant stress [82,83]. In order to observe the subtle changes in the strain of the different nanostructures of the experimental materials, the strain time diagrams of different specimens at the same temperature were compared. Figure 11 illustrates the strain changes in all three sets of specimens under three test temperatures. For samples sputtering at 75 W, it can be seen that when the creeping temperature was at 80 °C and 100 °C, the strain on specimen 1 barely changed with time. In contrast, thin films are deformed immediately at higher temperatures (120 °C) and gradually increase in deformation over time. In the test results of sample 2, the black line corresponds to the fitting curve of the raw data (red data) and showed a slight increase in creep strain at 100 °C and 120 °C. On the other hand, when creep testing is performed at 80 °C, the time has almost no effect on the results. Meanwhile, the strain on specimen 3 (125 W) at two different temperature sets (100 °C and 120 °C) creeps strongly upward with time. Similar to the other two samples tested at 80 degrees, we did not see a significant change in the strain during the creep test. Among them, higher creep temperature indicates a visible more time-dependent behavior than lower temperatures. Moreover, based on Figure 11, we can predict the creep equilibrium when all the samples at temperature have reached equilibrium when the creep test time is at 2.5 h.

## 5. Discussion

In this study, we tested MGTFs that have an amorphous structure. We observed that changing the sputtering power from 75 W to 125 W led to the formation of larger column sizes and a more irregular amorphous structure in the thin film. This may potentially result in defects or holes in the structure and also affected the modulus and hardness of the MGTFs. As seen in Figure 9, the cross-sectional image from the bottom revealed that the structure of the ZrCuAlNi thin metallic glass films transformed gradually. At 125 W, the thin film nearly became an irregular amorphous structure, which made it easier to deform, as indicated in the experimental results showing lower Young’s modulus and hardness. One may argue that the use of higher RF power during deposition could potentially result in increased thermal stress. This hypothesis is supported by the SEM image presented in Figure 9c, which shows evidence of such thermal stress, thereby affecting the residual stress in the component, as shown in Table 2. The use of an RF power source with higher power results in a film that is more disordered and amorphous than one produced using a lower-power source. This is because the more energetic collisions between the sputtering ions could lead to the atoms in the film being arranged more randomly.

The results of the SEM analysis of the ZrCuAlNi thin-film metallic glass reveal that the surface morphology is strongly influenced by the RF power used during the sputtering process. The observed changes in morphology are attributed to the elevated flux of particles with higher kinetic energy reaching the surface per unit of time. While the increase in atomic mobility at high-RF power levels is not enough to induce crystallization in this thin-film coating system, it does restrict diffusion and lead to the growth of more islands simultaneously, resulting in columns. Larger columnar structures with dimensions ranging from 20 to 40 nm were observed in our study, which is consistent with the value reported by Niklas [40]. The results suggest that controlling the RF power during the sputtering process is critical for obtaining the desired surface morphology in ZrCuAlNi thin films.

It was observed that ZrCuAlNi thin metallic glass films demonstrate substantial creep resistance at temperatures of 80 °C, 100 °C, and 120 °C when compared to thin metal films. The results of the creep test reveal that at a low temperature range of approximately 80 °C, the creep behavior of the 100 W and 125 W samples is found to be similar, which is reflected in the hardness outcomes. However, this is in stark contrast to the 75 W sample, which shows superior resistance to creep. On the other hand, at higher temperatures of 100 °C and 120 °C, a notable difference in strain is observed among the three tested samples. The strain progressively increases over time. This can be attributed to the amorphous structure of metallic glass films, which lack grain boundaries and grains, thus inhibiting the grain boundary slip, dislocation, and coble creep mechanisms observed in metals, thereby enhancing the anti-creep ability. The experimental results were compared to previous studies, which indicated that the metallic glass film is resistant to creep, with a better creep resistance than polycrystalline aluminum films. However, the comparison of the creep test results with previous literature [71] results on a pure metal, Al, raises questions on the comprehensiveness of the comparison. In order to address this, additional experiments were conducted to investigate the creep resistance of the ZrCuAlNi metallic glass film. The results obtained showed that the creep resistance of the ZrCuAlNi metallic glass film was better than that of pure aluminum films and different kinds of Al-Ti alloy films of polycrystalline structure, even at temperatures as low as 80 °C [84].

Moreover, since metallic glasses films do not have crystal grains and grain boundaries, the creep mechanism of metallic glasses is not easy to observe compared with general crystalline metals. Previous works of literatures [85,86] established a simulation model through nanoindentation and found that the creep mechanism of metallic glasses has an apparent relationship with atomic diffusion and the Shear Transformation Zone (STZ): the smaller the STZ volume, the lower the creep resistance [86,87]. The experimental method cannot directly measure the STZ volume change through the bulge system. However, it can be observed (in Figure 11) that the creep strain of specimens 2 and 3 tended to increase with the increase in higher temperature.

In Figure 11, it is evident that there is a fair amount of creep behavior with an addition of the RF power on sputtered films. Here, the temperature in the testing range of 80 to 120 °C is considered tested as the room temperature heated up to the temperature mentioned above [88]. According to the time and strain curves shown in Figure 11, temperature plays an important role in the identification of MGTF structures in creep experiments. There was a sharp strain observed in samples 2 and 3 due to stress, and Young’s modulus, as well as hardness, decreased with increasing experiment temperatures [89]. The observed creep phenomenon implies that amorphous structures may have formed in the studied alloys, which led to anisotropic mechanical properties. Specifically, the shear-band deformation observed in amorphous alloys, which corresponds to samples 2 and 3 in our study, along with the hardness of the MGTF of these materials, indicates that the amorphous structures present in these alloys have a significant impact on their mechanical behavior. These findings suggest further investigation into the role of amorphous structures in the mechanical properties of metallic alloys.

Zirconium-based MGTFs are prepared with three different sputtering powers. We can see that at the beginning of each sample at each higher temperature, creep strain will in turn be higher. This is consistent with the hardness measurements of each sample decreasing with increasing sputtering power. Compared with samples 2 and 3 deposited at higher sputtering powers, sample 1 with lower sputtering power shows better creep resistance and higher hardness.

## 6. Conclusions

The present study investigates the effects of sputtering power on the nanostructure, surface morphology, and mechanical properties of ZrCuAlNi MGTFs deposited using RF magnetron sputtering. The films were deposited at sputtering powers of 75 W, 100 W, and 125 W, and their nanostructure and surface morphology were characterized using XRD, SEM, and EDS. The mechanical properties of thin film were evaluated through bulge tests and nanoindentation, including Young’s modulus, residual stress, and hardness. To further investigate the behavior of thin film, additional heating sources will be added to the bulge tests to examine their creep resistance.

The results demonstrate that the sputtering power has a significant impact on the nanostructure, surface morphology, and mechanical properties of the thin films. Specifically, higher sputtering power results in surface bulging, while lower sputtering powers yield films with higher hardness. The hardness and Young’s modulus of the films were observed to decrease with increasing sputtering power. The films exhibited excellent creep resistance at temperatures of 80 °C, 100 °C, and 120 °C. Notably, the MGTF deposited at a sputtering power of 125 W exhibited weaker creep resistance than the other samples, whereas lower sputtering powers led to creep behavior with higher creep resistance.

This study underscores the significance of sputtering power in determining the nanostructure, surface morphology, and mechanical behavior of ZrCuAlNi MGTFs. The findings suggest that lower sputtering powers offer better creep resistance. Further investigations involving additional heating sources will provide a more comprehensive understanding of the mechanical behavior of the thin films.

## Figures and Tables

**Table 1:** Sputtering process parameters for the bulge specimen.

	Sample 1 (75 W)	Sample 2 (100 W)	Sample 3 (125 W)
Ambient pressure (Torr)	5 × 10^−6^	5 × 10^−6^	5 × 10^−6^
Argon flow (sccm)	22	22	22
Working pressure (Pa)	1	1	1
The target-to-substrate distance (cm)	15	15	15
RF power supply (W)	75	100	125
Sputtering rate (nm/min)	10.63	17.91	25.45
Duty cycle time (min)	9.4_on_/5_off_	5.6_on_/5_off_	3.93_on_/5_off_
Parity	5	5	5

**Table 2:** EDS data of atomic percentages and calculated ratios for thin metallic glass films.

Element	Target	Sample 1 (75 W)	Sample 2 (100 W)	Sample 3 (125 W)
Zr (%)	55	53.98 ± 3.58	54.64 ± 3.42	55.81 ± 3.32
Cu (%)	30	32.1 ± 3.21	31.34 + 3.15	30.33 ± 3.45
Al (%)	10	9.47 ± 1.43	9.31 ± 1.69	9.24 ± 1.64
Ni (%)	5	4.45 ± 1.04	4.71 ± 0.28	4.62 ± 0.26

**Table 3:** Measurement results on the mechanical properties of the bulge specimens.

	Sample 1 (75 W)	Sample 2 (100 W)	Sample 3 (125 W)
Bulge Young’s modulus (GPa)	84.25 ± 3.58	80.81 ± 3.88	78.65 ± 3.65
Nanoindentation Young’s modulus (GPa)	87.63 ± 3.72	83.16 + 3.99	80.43 ± 3.74
Bulge Residual stress (MPa)	33.65 ± 1.43	35.18 ± 1.69	13.73 ± 0.64
Nanoindentation Hardness (GPa)	6.29 ± 0.27	5.84 ± 0.28	5.62 ± 0.26

## Data Availability

Data sharing is not applicable to this article.
